# First custom next-generation sequencing infertility panel in Latin America: design and first results

**DOI:** 10.5935/1518-0557.20190065

**Published:** 2020

**Authors:** Daniela Lorenzi, Cecilia Fernández, Melina Bilinski, Mónica Fabbro, Micaela Galain, Sebastián Menazzi, Mariana Miguens, Pamela Nicotra Perassi, María Florencia Fulco, Susana Kopelman, Gabriel Fiszbajn, Florencia Nodar, Sergio Papier

**Affiliations:** 1 Novagen. Buenos Aires, Argentina; 2 Centro de Estudios en Genética y Reproducción (CEGYR). Buenos Aires, Argentina

**Keywords:** infertility, gene panel, next-generation sequencing, genetic variant

## Abstract

**Objective:**

To present the development of the first custom gene panel for the diagnosis of male and female infertility in Latin America.

**Methods:**

We developed a next-generation sequencing (NGS) panel that assesses genes associated with infertility. The panel targeted exons and their flanking regions. Selected introns in the *CFTR* gene were also included. The *FMR1* gene and Y chromosome microdeletions were analyzed with other recommended methodologies. An in-house developed bioinformatic pipeline was applied for the interpretation of the results. Clear infertility phenotypes, idiopathic infertility, and samples with known pathogenic variants were evaluated.

**Results:**

A total of 75 genes were selected based on female (primary ovarian insufficiency, risk of ovarian hyperstimulation syndrome, recurrent pregnancy loss, oocyte maturation defects, and embryo development arrest) and male conditions (azoospermia, severe oligospermia, asthenozoospermia, and teratozoospermia). The panel designed was used to assess 25 DNA samples. Two of the variants found were classified as pathogenic and enable the diagnosis of a woman with secondary amenorrhea and a man with oligoasthenoteratozoospermia. Targeted NGS assay metrics resulted in a mean of 180X coverage, with more than 98% of the bases covered ≥20X.

**Conclusion:**

Our custom gene sequencing panel designed for the diagnosis of male and female infertility caused by genetic defects revealed the underlying genetic cause of some cases of infertility. The panel will allow us to develop more precise approaches in assisted reproduction.

## INTRODUCTION

Infertility is a disease of the reproductive system characterized by the failure to establish a clinical pregnancy after 12 months of regular, unprotected sexual intercourse or due to an impairment of a person's capacity to reproduce either as an individual or with his/her partner (Zegers-Hochschild et al., 2009, 2017). Infertility has been described as a global public health issue not recognized as a priority to treat and resolve (Macaluso et al., 2010). Evidence estimates that 48.5 million couples worldwide are affected by infertility (Mascarenhas et al., 2012). The prevalence is estimated at around 9% (Boivin et al., 2007).

Infertility is generally diagnosed by clinical manifestations. In cases where an etiology is identified, it may be caused by female factors (~30%), male factors (~30%), or a combination of both (~40%). However, there are still many unexplained cases of infertility, classified as "idiopathic infertility" (~30%) (Lindsay & Vitrikas, 2015). Infertility is a pathology with a complex multifactorial etiology including environmental and genetic factors. It is estimated that these genetic factors are responsible for up to 50% of the infertility cases, so half of them still remain unexplained. There are hundreds of genes that need to be correctly expressed along the hypothalamic-pituitary-gonadal axis for the adequate performance of human reproductive system. The large number of candidate genes makes it difficult to find a genetic cause of infertility in the majority of the cases (Zorrilla & Yatsenko, 2013). With the advent of new DNA sequencing technologies and bioinformatic approaches, there have been huge research advances in understanding the genetic causes of infertility (Harper, 2018).

The genetic alterations that can have an impact on fertility are numerical or structural chromosomal aberrations and gene disorders (Jedidi et al, 2018). Epigenetics likely has a role in the pathogenesis of infertility as well, but its contribution is poorly understood (Comhaire et al., 2017). The algorithm for genetic testing in both male and female infertility starts with standard karyotyping of the affected couple (Harper, 2018).

There is a distinction between syndromic and non-syndromic infertility in cases where a monogenic alteration is detected. In the first case, infertility is not the main manifestation of the syndrome. In non-syndromic cases, on the other hand, gene mutations can cause fertility problems with no other clinical phenotype (Okutman, 2018).

Chromosomal alterations are found in about 7% of men with spermatogenic failure. Klinefelter syndrome (which consists in the presence of at least an additional X chromosome in a male; such as 47,XXY karyotype for the classical variant) is the most common genetic cause of azoospermia, representing 14% of the cases (McLachlan & O'Bryan, 2010). Y chromosome microdeletions are found in 10% of males with nonobstructive azoospermia (NOA) and 5% of men with severe oligospermia (Hotaling, 2014). Mutations in the *CFTR* gene, which causes cystic fibrosis (CF) and *CFTR*-related disorders, are the first cause of congenital bilateral absence of the vas deferens (CBAVD) and, therefore, obstructive azoospermia (OA) (Jedidi et al, 2018).

Regarding chromosomal abnormalities responsible for female infertility, several defects involving X chromosome have been associated with primary ovarian insufficiency (POI). The most frequent examples are Turner syndrome (45,X), with an incidence of 1 in 2500 women, and trisomy X (47,XXX), present in 1 of 1000 women (Cordts et al, 2011). Moreover, women who carry a premutation-level expansion of CGG repeats in the non-coding region of the *FMR1* gene (between 55 and 200 CGGs) have an increased risk of POI, as well as a high risk of having an affected child with Fragile X syndrome. It is estimated that harboring such molecular anomaly is associated with 11% risk of POI (Pastore & Johnson, 2014), whereas risk in general population is 1% (Coulam et al., 1986). However, the majority of POI cases are idiopathic (50-80% of cases). Non-syndromic POI is a complex pathology caused by low-to-drastic mutations in genes such as the ones that code for gonadotropins receptors. Additionally, several studies have described female infertility as a polygenic disease (Laissue, 2015).

The study of infertility caused by single-gene mutations is a rapidly changing field. In the past decade, several reports were published demonstrating the association of various genes with infertility (Ben Khelifa et al.,2012; Caburet et al., 2014; Ellnati et al., 2016; Amiri-Yekta et al., 2016; Desai et al., 2017). Initially, molecular diagnosis of infertility was carried out by the evaluation of a candidate gene that can explain a phenotype. This approach was time-consuming because of the hundreds of genes expressed along human sexual development. For instance, genes participating in sex determination, gametogenesis, hormonal cycle, fecundation and embryo development. High throughput sequencing technologies enable the simultaneous evaluation of multiple genes to search for genetic variants that may explain the complexity of infertility (Okutman, 2018; Laissue, 2015).

In this study, we present a panel designed to evaluate genetic causes of infertility using a next-generation sequencing (NGS) based approach, an in-house developed bioinformatic pipeline and an interdisciplinary interpretation of results.

## MATERIALS AND METHODS

### Patients

Individuals with already known pathogenic variants were included. Patients with a precise infertility phenotype that could benefit from the study were also recruited, as well as couples with unexplained ("idiopathic") infertility. Exclusion criteria were individuals with abnormal karyotypes or cases in which infertility was a secondary finding within a monogenic syndrome that severely affects other organs or systems. For the *FMR1* CGG repeat region and Y chromosome microdeletion analysis samples with known normal and pathogenic genotypes were also used for the validation set.

### Samples DNA isolation

DNA was extracted from blood and saliva using the QIAamp Mini Kits (Qiagen) following the manufacturer's instructions. DNA was quantified using Qubit dsDNA BR Assay Kit and the Qubit^®^ 3.0 fluorometer. The Speed-Vac concentrator was used to concentrate samples if it were necessary.

### Custom panel design

Genes with proven disease-associated variants related to infertility disorders were included in the targeted NGS panel ("diagnostic genes"). The panel also included "candidate genes" which their association with infertility needs further study.

The selection of the genes was based mainly in relevant literature referenced in PubMed and Online Mendelian Inheritance in Man (OMIM) and guided by ClinGen Clinical Validity Classifications (Strande et al., 2017).

The target panel included all the coding exons and flanking regions of at least 10 nucleotides upstream and downstream of each exon (based on RefSeq database). Clinically relevant noncoding (intronic) regions that contained previously described pathogenic variants in the *CFTR* gene were also included. In addition, selected regions for the detection of the gender of the sample were included as an internal control.

### Next-generation DNA sequencing

DNA samples were prepared for sequencing following the manufacturer's instructions described in the SureSelectQXT Target Enrichment for Illumina Multiplexed Sequencing manual (Agilent Technologies, Inc.), a transposase-based library preparation technology. DNA libraries quantification was performed using Qubit dsDNA Assay with the Qubit^®^ 3.0 fluorometer. DNA libraries quality and integrity were assessed using the Agilent 2100 Bioanalyzer (Agilent Technologies, Inc.). DNA Libraries were sequenced on a MiSeq platform (Illumina, San Diego, CA) using a 300 cycles-MiSeq Reagent Micro Kit v2 following the manufacturer's instructions.

### Sequence analysis and variant interpretation

Sequence data analysis was carried out using an in-house developed bioinformatic pipeline, built on the best practices in the field. First, data were demultiplexed and converted to Fastq format simultaneously using bcl2fastq software (Illumina, 2017). Quality control of the data was performed using FastQC software (Babraham Bioinformatics, 2017). Reads were aligned against the human reference genome hg19 using Burrows-Wheeler Aligner v0.7.17 (Li & Durbin, 2009) and in order to detect single-nucleotide variants (SNVs) and small insertions and deletions, Genome Analysis Toolkit v4.0 was used (Van der Auwera et al., 2013). Then, for obtain biological and functional information, variants were annotated against several databases, such as ClinVar, dbSNP, 1000Genomes, dbNSFP and ExAC, among others, using SnpEff software v4.3i (Cingolani et al., 2012).

We used allele frequency to classify all detected variants as common or rare. Variants with an allele frequency ≥ 5% in ExAC were defined as common variants and were filtered out, with some exceptions. Several of the included genes were related to pharmacogenetic effects. Specifically, genes encoding receptors that may alter the response to treatment for ovarian hyperstimulation were analyzed. For those genes, variants with higher allele frequency than 5% were evaluated as well. In addition, the results of the different bioinformatic predictors of pathogenicity and the type of effect of the variant were taken into account for the filtering process.

Finally, filtered variants were classified as pathogenic, likely pathogenic, variant of unknown significance (VUS), likely benign or benign, according to the American College of Medical Genetics and Genomics (ACMG) and the Asso¬ciation for Molecular Pathology (AMP) guidelines (Richards et al., 2015). The report of the variants was made according to the Human Genome Variation Society (HGVS) nomenclature (den Dunnen et al., 2016).

#### *FMR1* testing

*FMR1* testing was performed using 20-80 ng of genomic DNA per sample. The *FMR1* CGG repeat region was amplified using the AmplideX^®^ PCR/CE FMR1 kit (TP-PCR) (Asuragen, Austin, TX), following the manufacturer's instructions. PCR products were then separated by capillary electrophoresis on an ABI 3500xL (Macrogen, Inc., Korea). Manual annotation was performed using GeneMapper^®^ 5.0 software (Applied Biosystems^TM^).

#### *CFTR*, T/TG tract analysis

The amplification of intron 9 - exon 10 region was performed using the following conditions. Briefly, genomic DNA was amplified in a reaction that contained 2µL 50µM of each primer (Invitrogen), 0.4µL 10 mM dNTP Set (Invitrogen), 2,5µL 50 mM MgCl, 0,2µL 5U/µL Taq DNA polymerase (Invitrogen), 5µL 10x buffer and 2µL 20-60 ng/µL genomic DNA. It was completed with sterile distilled water up to a volume of 50µL. The primers used were: 5´CCATGTGCTTTTCAAACTAATTGT3´ (forward), 5´TAAAGTTATTGAA TGCTC GCCATG 3´(reverse).

PCR was performed using predefined reaction conditions: 94°C for 3 min; 30 cycles of 94°C for 30 sec, 55°C for 30 sec, 72°C for 1 min; and 72°C for 5 min. PCR products were analyzed using agarose gel electrophoresis to confirm appropriate amplification. Purified PCR products were directly sequenced using the 3730XL automated DNA sequencer (Macrogen, Inc, Korea). Results were analyzed with FinchTV software V1.4.0 (Geospiza Inc., Seattle, WA) using the sequence NM_000492 as a reference (RefSeq database).

### Y chromosome microdeletion analysis

The diagnosis of Y chromosome microdeletion was made by two multiplexes PCRs. Both reactions amplify three AZF loci and the SRY control fragment. DNA samples from a fertile male and female were used as an internal controls in each multiplex assay according to the recommendations of the European Academy of Andrology (EAA) and the European Molecular Genetics Quality Network (EMQN) guidelines (Krausz et al, 2014).

The 50 µL PCR reaction mix contained: 0.4µL 10 mM dNTP Set (Invitrogen), 1.85µL 50 mM MgCl, 0.4µL 5U/µL Taq DNA polymerase (Invitrogen), 2.5µL 10x buffer, 4µL 100 ng/µL template DNA and sterile distilled water.

Amplification conditions start with an initial step of 94°C 2 min, followed by 35 cycles at 94°C for 30 sec, 60°C for 30 sec and 72°C for 30 sec, ended by a last elongation step of 7 min at 72ºC using a Veriti 96-well thermal cycler (Applied Biosystems, Foster City, CA). Reaction products were separated by 3% agarose gel electrophoresis.

### Sanger Sequencing

Pathogenic and likely pathogenic variants identified by NGS were validated using Sanger sequencing. First, the region of interest was amplified by PCR, following the appropriate conditions for each amplicon. PCR products were analyzed using agarose gel electrophoresis to confirm amplification and then quantified using Qubit dsDNA BR Assay Kit and the Qubit^®^ 3.0 fluorometer. Purified PCR products were sequenced following the Big Dye terminator sequencing protocol (Applied Biosystems, Foster City, CA, USA) and analyzed using a 3500XL capillary electrophoresis (Macrogen, Inc, Korea). Sequence analysis was performed using FinchTV software V1.4.0 (Geospiza Inc., Seattle, WA), and the reference sequence of RefSeq database.

## RESULTS

### Fertility gene panel design

After the selection and classification of genes associated with infertility, a custom sequencing panel was designed including 75 genes. Genes were grouped into two sub-panels: a female fertility panel and a male fertility panel (Table 1).

**Table 1:** Genes associated with female and male infertility included in the NGS panel. Genes in bold font have proven associations with infertility (diagnostic genes). Candidate genes are shown in normal font.

FEMALE CONDITIONS		MALE CONDITIONS	
Primary ovarian insufficiency	***BMP15, ESR1, FGFR1, FIGLA, FMR1, FOXL2, FSHB, FSHR, GALT, GDF9, HFM1, KISS1R, LHB, MCM8, MCM9, NOBOX, NR5A1, POF1B, PSMC3IP, SOHLH1, STAG3, SYCE1,** DIAPH2, DMC1, FOXO3, LHCGR, LHX8, MSH4, NANOS3, PGRMC1, REC8, SMC1B*	Non-obstructive azoospermia/ Severe oligospermia	***AR, AZF, FGFR1, FSHB, KAL1/ANOS1, KISS1R, KLHL10, LHB, NANOS1, NR5A1, SOHLH1, SYCE1, SYCP3, TAF4B, TEX11, TEX15, USP9Y, ZMYND15, ** HSF2 *
Oocyte maturation defects	***TUBB8, ZP1***	Obstructive azoospermia	***ADGRG2, CFTR***
Embryo development arrest	***PADI6, TLE6***	Oocyte activation failure	***PLCZ1***
Ovarian hyperstimulation syndrome	***FSHR, ** AMH, AMHR2, CAPN10, DENND1A, GDF9 LHCGR, SULT2A1, THADA *	Asthenozoospermia	***CATSPER1, DNAH1, DNAH5, DNAI1 SEPT12, SLC26A8, SUN5, *** *CATSPER2 GALNTL5 *
Recurrent pregnancy loss	*F2, F5, MTHFR, PROC, PROS1 SERPINC1, SYCP3*	Sperm morphology alterations	***AURKC, DPY19L2, SEPT12 SPATA16, SUN5,*** *PICK1, ZPBP *
	Hormone receptors*	***AR, ESR1, FSHR, LHCGR***	

* Hormone receptors are evaluated in both panels.

The female fertility panel included 50 genes associated with POI, risk of ovarian hyperstimulation syndrome, recurrent pregnancy loss, oocyte maturation defects and embryo development arrest. The *FMR1* gene was evaluated by TP-PCR as previously described.

The male fertility panel included 35 genes and was designed for the evaluation of patients with altered semen analysis: OA or NOA, low sperm count (severe oligospermia), alterations in sperm motility (severe asthenozoospermia) and anomalies in sperm morphology (teratozoospermia). In addition, it included a gene associated with oocyte activation failure. Evaluation of the AZF region was carried out by another methodology, as previously described.

As mentioned before, some genes were considered "candidate genes" because further research is necessary to clearly determine their association with different infertility phenotypes (Table 1).

Regarding the results of the NGS assay, the total size of the gene panel was 400 Kbp approximately, all the regions of interest were captured. The targeted NGS assay metrics resulted in a mean of 180X coverage, with more than 98% of the bases covered ≥20X.

#### *FMR1* testing

**Figure 1:**
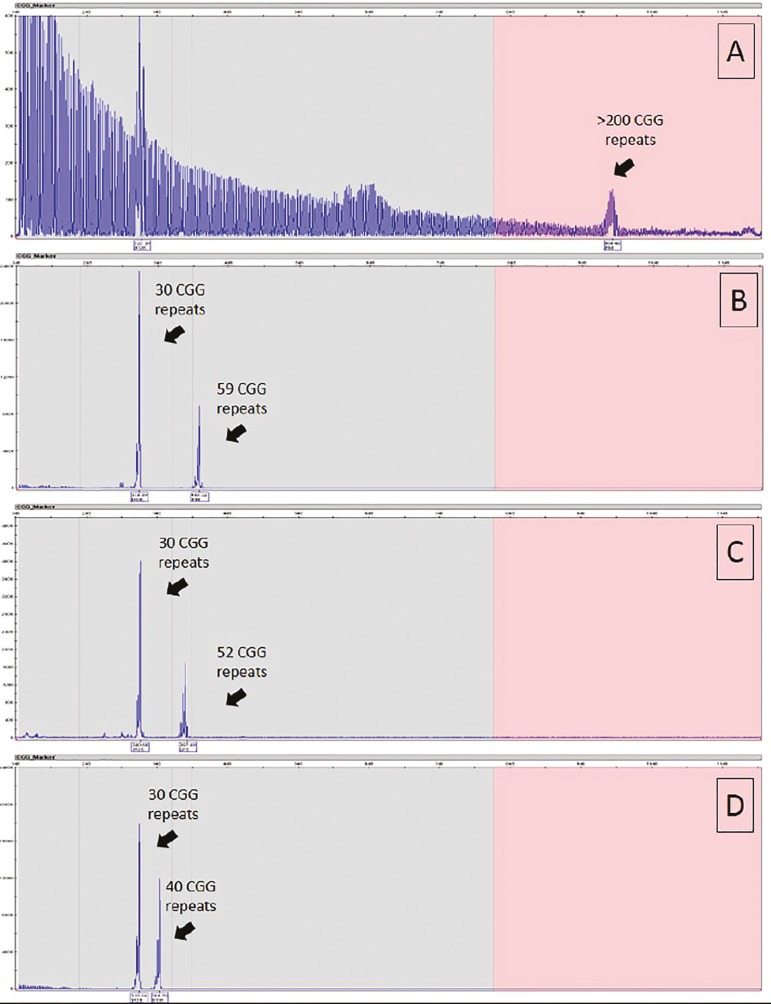
Fragile X testing. The number of CGG repeats was determined by Tripled Repeat Primed PCR amplification of the 5' untranslated region of the *FMR1* gene followed by capillary electrophoresis (AmplideX *FMR1* PCR Kit, Asuragen, Austin, TX, USA). (A): Patient with a full mutation, confirming the diagnosis of Fragile X syndrome; (B): Patient with two alleles with 30 and 59 CGG repeats, suggestive of premutation; (C): Patient with two alleles with 30 and 52 CGG repeats, which corresponds to an “intermediate allele” carrier; (D) Patient with two normal alleles with 30 and 40 CGG repeats

To complement the infertility genetic panel with *FMR1* study, we used an already available protocol based on PCR amplification and capillary electrophoresis (Filipovic-Sadic et al., 2010). We correctly identified all alleles previously classified as "mutation" (> 200 CGG repeats), "pre-mutation" (55 to 200 CGG repeats), "intermediate" (45 to 54 CGG repeats) and "normal" (<45 CGG repeats) (Figure 1) in the control samples.

### Y chromosome microdeletion analysis

**Figure 2:**
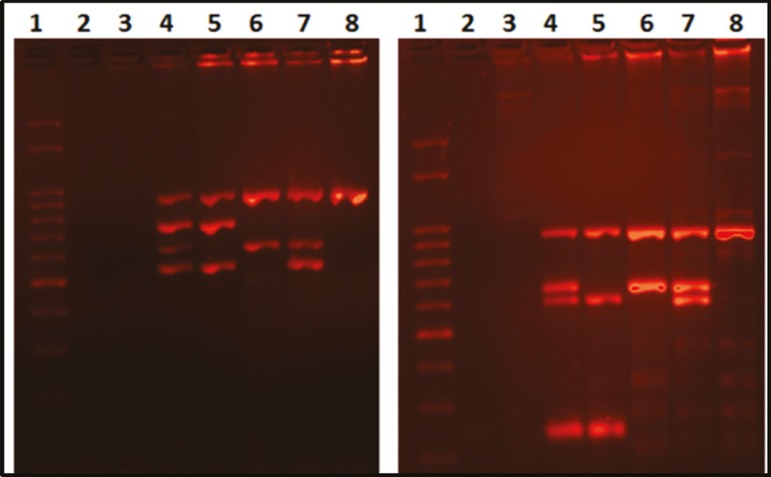
Y chromosome microdeletion analysis. Examples of both multiplex PCR. Lane 1: marker 50pb, lane 2: water, lane 3: female DNA, lane 4: DNA of a normal male, lane 5: DNA of AZFa deleted patient, lane 6: DNA of AZFbc deleted patient, lane 7: DNA of AZFc deleted patient and lane 8: DNA of AZFabc deleted patient

Diagnostic testing for deletions was performed by PCR amplification of selected regions of the Y chromosome. The STS (Sequence Tagged Sites) primers used have been shown to give robust and reproducible results in multiplex PCR reactions by several laboratories (Krausz et al. 2014). Each set of PCR reactions were carried out by two separated multiplex PCR reactions in order to distinguish a negative result from a technical failure. Microdeletions were correctly identified in the positive control samples (Figure 2).

The methodology was fast, cost-effective and it represents the gold standard for the detection of Y chromosome microdeletions.

#### *CFTR*, T/TG tract analysis

A string of thymidine bases located in intron 9 of *CFTR* can be associated with *CFTR*-related disorders, depending on its size. The three common variants of the poly T tract are 5T, 7T, and 9T. Both 7T and 9T are considered benign variants and 5T (commonly referred to as the 5T allele) is considered a disease susceptibility variant with variable penetrance. The 5T allele is thought to decrease the efficiency of intron 9 splicing (Chu et al., 1993; Hefferon et al., 2002).

A TG tract lies just 5' of the poly T tract. It consists of a short string of TG repeats that commonly number 11, 12, or 13. Longer TG repeat sizes (12TG and 13TG) in *cis* with 5T allele are associated with a greater susceptibility to disease than 5T allele in *cis* with a smaller TG repeat size (11TG) (Cuppens et al., 1998; Groman et al., 2004).

**Figure 3:**
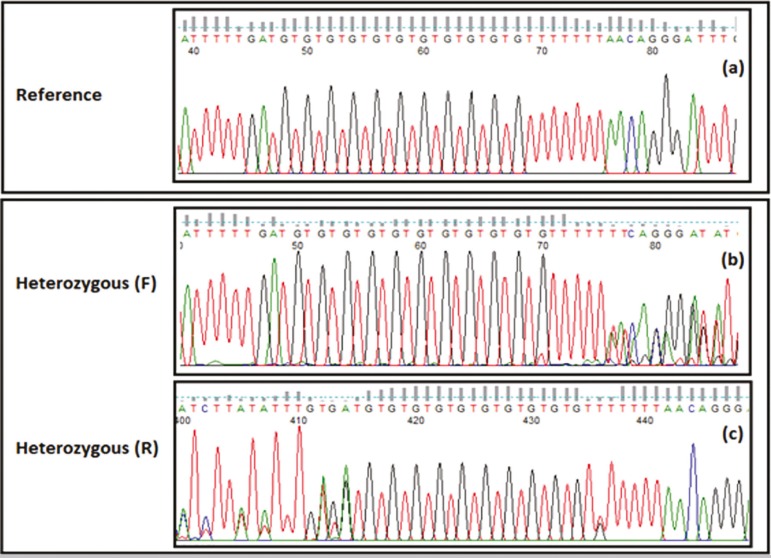
Sanger sequencing results for TG/T tract in **CFTR**. a: sample homozygous for 11TG-7T b,c: sample heterozygous for 11TG-5T and 11TG-7T. F: forward primer; R: reverse primer

To determine the number of both tracts, direct Sanger sequencing of the region was used (Figure 3). The expected genotypes were obtained for the control samples, and no nonspecific amplification was detected. The number of T and TG repeats could be determined accurately.

### Results from infertile individuals

The panel was tested with 25 samples: 11 patients with POI, 1 patient with secondary amenorrhea, 2 women with recurrent pregnancy loss, 1 sample for embryo arrest indication, 2 female samples with idiopathic infertility, and 4 male samples presenting severe asthenozoospermia, oligoasthenoteratozoospermia and 2 with NOA. Two male samples with known variants in *CFTR* were included as positive controls, as well as 2 healthy individuals. Genetic results are described in Table 2.

**Table 2:** Genetics variants found in the analysis of 25 samples.

Sample	Gender	Phenotype	Gene	Nucleotide variant	Amino acid variant	Category
1	F	POI	*MCM8*	c.-5-7C>T	-	VUS
2	F	Secondary amenorrhea	*GDF9*	c.1360C>T	p.Arg454Cys	Likely pathogenic
3	F	POI	*MCM9*	c.3223C>T	p.Pro1075Ser	VUS
			*STAG3*	c.1467+3G>A	-	VUS
4	F	POI	*SOHLH1*	c.835G>A	p.Glu279Lys	VUS
5	F	POI	*-*	-	-	-
6	M	Male control	*-*	-	-	-
7	F	POI	*-*	-	-	-
8	F	Female control	*-*	-	-	-
9	M	*CFTR* control	*CFTR*	c.1521_1523delCTT	p.Phe508del	Pathogenic
			*CFTR*	c.3718-2477C>T	-	Pathogenic
10	M	*CFTR* control	*CFTR*	5T allele ^†^	-	*
11	M	Severe asthenozoospermia	*-*	-	-	-
12	F	POI	*-*	-	-	-
13	F	POI	*F2*	c.*97G>A	-	Pathogenic
			*REC8*	c.492_512delAGAGAGAGTTGAAGAGATCCC	p.Glu165_Pro171del	VUS
14	F	POI	*FOXO3*	c.76A>T	p.Ser26Cys	VUS
			*FOXO3*	c.246_251delCGGCGG	p.Gly83_Gly84del	VUS
15	F	POI	*-*	-	-	-
16	F	POI	*-*	-	-	-
17	F	Recurrent pregnancy loss	*CFTR*	5T allele ^†^	-	-
18	F	Recurrent pregnancy loss	*F2*	c.598G>A	p.Glu200Lys	VUS
19	M	NOA	*CFTR*	5T allele ^†^	-	-
20	F	POI	*STAG3*	c.1640A>G	p.Glu547Gly	VUS
21	F	Embryo arrest	*CFTR*	5T allele ^†^	-	-
22	F	Idiopathic infertility	*F2*	c.*97G>A	-	Pathogenic
23	M	Oligoasthenoteratozoospermia	*AR*	c.2395C>G	p.Gln799Glu	Likely pathogenic
24	F	Idiopathic infertility	*CFTR*	c.224G>A	p.Arg75Gln	VUS
			*CAPN10*	c.1663C>T	p.Arg555Cys	VUS
25	M	NOA	*NANOS1*	*c.100C>A*	p.Pro34Thr	VUS

† c.1210-7_1210-6delTT, commonly known as 5T allele.

VUS: variant of uncertain significance

Two pathogenic variants were identified. The variant c.1360C>T (p.Arg454Cys) in the *GDF9* gene was detected in a young female with secondary amenorrhea. Heterozygous mutations in this gene were associated with this phenotype and POI (Dixit et al., 2005; Laissue et al., 2006; Kovanci et al., 2007). Bioinformatic predictors of pathogenicity predicted a harmful effect on the protein function. This variant was also described in patients with primary amenorrhea, together with another mutation in the *REC8* and *FIGLA* genes. These findings led the authors to propose that POI could be a digenic pathology in some cases (Bouilly et al., 2016). Considering *in-silico* pathogenicity predictions and population frequency, the variant was classified as likely pathogenic using ACMG guidelines.

Variant c.2395C>G (p.Gln799Glu) in the *AR* gene was detected in a man with altered spermogram studies (oligoasthenoteratozoospermia) who started assisted reproduction treatments in 2012. Mutations in the *AR* gene cause complete, partial or mild androgen insensitivity syndrome depending on the type and the location of the mutation in the gene. Patients with the mild form, like this case, may present infertility as their only symptom. This variant has also been described in patients with alterations in spermatogenesis such as azoospermia and oligoteratozoospermia (Bevan et al., 1996; Hiort et al., 2000). It was also classified as likely pathogenic according to all the information available.

Regarding the *CFTR* control samples, sample 9 carried the p.Phe508del variant (the most common *CFTR* mutation) and an intron variant (c.3718-2477C>T), and sample 10 presented the 5T allele in intron 9 (c.1210-7_1210-6delTT). All the mutations were identified by our methodology. These results corroborate the capacity of the designed panel for the detection of relevant *CFTR* variants in coding and intronic regions of interest.

5T allele in the *CFTR* gene was identified in 3 samples. As previously mentioned, the number of the TG repeats in *cis* should be tested additionally in order to determine the possible severity. Allele 11TG was determined by Sanger sequencing in *cis* with the 5T allele in all cases. This allele combination is associated with a milder severity.

The pathogenic variant identified in the *F2* gene (c.*97G>A) is the most frequent mutation in this gene. It has been associated with a higher susceptibility to recurrent pregnancy loss. This finding warrants further study with hematologic tests in order to identify thrombophilia and decide the ideal therapeutic approach accordingly.

Twelve variants in genes related to the phenotype of the patients were classified as VUS according to the information available. Even though a VUS should not be used for a clinical decision-making, the ACMG recommends the report of VUS in genes related to a clinical indication (Rehm et al., 2013), since future research might allow the reclassification of the VUS as a pathogenic or benign variant.

## DISCUSSION

Over the last years, the advent of high throughput DNA sequencing methods and bioinformatics has allowed a better understanding of the genetic basis of diseases in many fields of medicine. Infertility is a complex multifactorial disease reflected by the broad spectrum of clinical manifestations and heterogenetic aspects. Hundreds of genes have to interact in a precise manner in order to a healthy child to be born. The genetics of infertility has advanced slowly compared to other diseases. Nevertheless, several genes have unquestionably been associated with infertility disorders.

Whole Exome Sequencing (WES) has allowed the identification of a huge number of genetic defects associated with a variety of diseases. Studies of large cohorts of infertile patients by WES will enable the identification of new genes related to infertility in the very near future. However, WES approach is not always feasible in the clinical setting, taking into account financial limitation in some countries and the challenging and time-consuming analysis and interpretation of the results.

For clinical application, gene panels permit high-throughput sequencing of a predefined list of genes, reducing costs and saving time. Fertility panels, like the one we present here, seek to identify genetic variants that may explain the cause of male and female infertility. Besides, custom gene panels have the advantage that can be modified over time, adding new genes discovered or removing genes that were finally not suitable for the diagnosis of infertility. Therefore, these studies have the potential to expand the knowledge about genetics and infertility.

In our design, we include diagnostic genes, with largely demonstrated clinical implication in infertility and candidate genes, whose associations with infertility still required further elucidation.

Our genetic infertility panel relies on several methodologies, such as PCR, TP-PCR, NGS and Sanger sequencing, in order to obtain a comprehensive test of the causes of infertility. The use of the recommended and extensively proved methodologies for the *FMR1* CGG repeat region and the Y chromosome microdeletions analysis provides reliable results.

Y-chromosomal microdeletions are the second most frequent genetic cause of male infertility after Klinefelter syndrome. Thus, testing the AZF region is mandatory in male patients with NOA or severe oligozoospermia.

The STS primers panel used are derived from non-polymorphic regions of the Y chromosome which are well-known to be deleted in men affected by oligo-/azoospermia. Additionally, the analysis of two STS loci in each region reinforces diagnostic accuracy. The methodology we used in this panel has been recommended for routine diagnostics and enables the detection of almost all clinically relevant deletions and over 95% of AZF deletions reported in the literature (Krausz et al. 2014).

There are two reasons for including analysis of the *CFTR* gene in the fertility panel. As mentioned above, mutations in this gene are the first cause of CBAVD in men. Additionally, the high prevalence of *CFTR* mutations for cystic fibrosis in our population and the severity of this disease make it important to detect couples who may be *CFTR* carriers and therefore, prevent the birth of a child affected by severe phenotypes. Upon written consent, women who underwent the female fertility panel and men who do not present OA can learn about their carrier status for *CFTR* mutations.

The 5T allele has been associated with recessive *CF*-related disorders when detected in *trans* with another pathogenic variant in the *CFTR* gene. Disease features include CBAVD in males and non-classic CF, depending on the variant on the opposite allele and the number of TG repeats (Chillon, 1995). The combination of the 5T allele and 11TG in *cis* is likely to exhibit a normal phenotype. However, individuals with 5T adjacent to either 12 or 13 TG repeats are substantially more likely to exhibit an abnormal phenotype (Cuppens, 1998; Groman, 2004). Thus, determination of the number of T and TG repeats is necessary for an accurate prediction of the pathogenic degree of 5T alleles.

The bioinformatic alignment of the reads in the poly-T region of the *CFTR* gene obtained by NGS is challenging, as there are variable numbers of poly-T and TG repeats in each individual allele. Most available NGS softwares cannot align this region properly. Sanger sequencing is still the gold standard for many determinations in the clinical setting. Our results show its robustness, high accuracy and specificity to characterize the poly-T region.

POI is one of the main causes of female infertility. A genetic cause of POI has been well-established in women carrying the fragile X premutation (Qin et al., 2015). Up to 20% of female *FMR1* premutation carriers develop POI. Of those, 1/3 will experience cessation of menstrual cycle at or before 29 years (De Caro et al., 2008). Female *FMR1* premutation carriers must be counseled about the high risk of having an affected male child with the physical and behavioral features of Fragile X syndrome, and about the available methods for preventing its occurrence, such as preimplantation genetic testing.

The ACMG has issued a policy statement recommending Fragile X testing for "women with reproductive or fertility problems associated with elevated FSH levels, especially if there is a family history of premature ovarian failure, Fragile X syndrome, or undiagnosed mental retardation" (Monaghan et al., 2013; Sherman et al., 2005). The Genetics Committee of the American College of Obstetrics & Gynecology supports the ACMG recommendation (ACOG Committee on Genetics, 2010).

Currently, there are no successful therapies to regain ovarian function in women with POI. However, hormone replacement therapy and fertility preservation options are available for women with an increased risk of POI or with signs of rapidly declining ovarian reserve.

The fertility panel designed includes genes that code for hormones and hormonal receptors which participate in the human reproductive system. Several studies have shown the relationship between certain polymorphisms in genes that encode receptors, such as the ones that bind to FSH and LH, with the outcome after a controlled ovarian hyperstimulation cycle and *in vitro* fertilization treatment (Altmäe et al., 2011; Alviggi et al., 2018). Results from the designed genetic panel will provide the necessary information to establish the prevalence of these polymorphisms in our population and determine its utility in the clinical practice.

It is important to highlight the role of clinicians who request this genetic study. Clinicians need to have a clear understanding of the phenotype of the patient to determine if the gene panel can identify the underlying cause of infertility. Many patients suffer from "idiopathic infertility", and even though in several cases a genetic cause may be found, the lack of a clear phenotype may represent a limitation for the interpretation of data, especially variants of uncertain significance. It is also important for clinicians to understand that interpreting this kind of studies is heavily influenced by three components: phenotypic features of the individual, medical history, and relevant family history. It is essential to report all observable features and the medical history of the family to the diagnostic laboratory to ensure proper interpretation of variants identified through testing (Normand et al., 2018).

Interpretation of VUS represents a difficult challenge. However, it is important to identify and reevaluate them in the future because, as research in the reproductive field advances, VUS that are currently not clearly associated with a phenotype may be classified as pathogenic or dismissed as benign.

There is a term increasingly used named "precision medicine" that seeks the best disease treatment and follow-up taking into account individual variability in genes, environment, and lifestyle for each person. This approach will allow physicians and researchers to predict more accurately which strategies will work in each group of people for a particular disease (https://ghr.nlm.nih.gov/primer/precisionmedicine). A fundamental requirement for this approach is the understanding of the genetic background of the patient and how certain genetic variants interact with the environment to affect individual health. In this context, genetic studies will be indispensably required for future precision medicine.

As more genes are discovered and the etiology of infertility disorders becomes better understood, the management and treatment of infertility will likely improve as well. Besides, genetic counseling will become increasingly important, especially in patients presenting a genetic cause of infertility and for future parents to make informed reproductive choices.

## CONCLUSION

Multi-disease gene panels have demonstrated an improvement in the clinical diagnosis of many diseases. The intersection of assisted reproductive technologies and genomics is a fast-growing scientific field. The increasing knowledge about the genetic background of individuals, together with rapid technological developments, have fostered improved diagnostics in infertility. Nowadays, in addition to the current routine testing of patients, selected gene panels are a useful tool for the identification of genetic causes of infertility.

Here, we present the first custom gene sequencing panel in Latin America, designed for the diagnosis of male and female genetic infertility. The application of this panel will improve the understanding of the genetic basis of infertility, improve genetic and reproductive counseling and ultimately, achieve more precise approaches in assisted reproduction.
